# Cell spheroids culture array with modifiable chemical gradients

**DOI:** 10.1111/cpr.13473

**Published:** 2023-05-17

**Authors:** Panhui Yang, Lei Wu, Guoyuan Zhang, Yuqing Ge, Ting Liu, Hongju Mao, Jianlong Zhao

**Affiliations:** ^1^ Shanghai Institute of Microsystem and Information Technology, CAS Shanghai China

## Abstract

Cancer cell spheroids have been shown to mimic in vivo tumour microenvironment and are therefore suitable for in vitro drug screening. Microfluidic technology can provide conveniences for spheroid assays such as high‐throughput, simplifying manual operation and saving reagent. Here, we propose a concentration gradient generator based on microfluidic technology for cell spheroid culture and assay. The chip consists of upper microchannels and lower microwells. After partitioning HepG2 suspension into the microwells with concave and non‐adhesive bottoms, spheroids can spontaneously form. By controlling the fluid replacement and flow in microchannels, the doxorubicin solution is diluted automatically into a series of concentration gradients, which spanning more than one order of magnitude. And then the effect of doxorubicin on spheroids is measured in situ by fluorescent staining. This chip provides a very promising approach to achieve the high‐throughput and standardized anti‐cancer drug screening in future.

## Graphical abstract

We propose a concentration gradient generator for cell spheroid culture and drug screening. The chip structure is designed with the guidance of fluid simulation. With the PDMS concave‐bottom microwell structure, cell spheroid formation is achieved inside the chip (step 1). To test the effect of drug on the spheroids, a section of drug solution is diluted gradually when flowing over the microwells in sequence and then a concentration gradient forms in the microwells along the microchannel (step 2). After drug treatment, the cell viability is evaluated with fluorescent staining in situ (step 3).

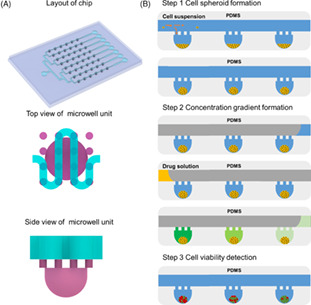

## 1 INTRODUCTION

Cell spheroids are three‐dimensional (3D) aggregations of cells. The compactness nature of cell spheroids endows them with some characteristics similar to tumour tissue in vivo.^1^, ^2^ Artificial in vitro cell spheroids provide similar metabolism and proliferation as in vivo. These properties make cell spheroids a more suitable tool for in vitro drug screening analysis than two‐dimensional (2D) monolayer cell cultures.^3^ For example, cancer cell spheroids have been shown to mimic in vivo tumour microenvironment and are therefore suitable for in vitro drug screening.

For cell culture, the traditional method of 96/384‐well plates is labour‐intensive. In contrast to traditional methods, microfluidic platforms can be used for long‐term perfusion cell culture while maintaining high cell viability. Given its permeable nature, PDMS has been proven to be ideal for cell spheroid formation and long‐term perfusion culture. Culturing cells and performing drug screening based on microfluidic chips is able to simplify the processes of cell loading, dispensing, medium exchange, and fluorescent labelling, and hopefully reduce the consumption of relevant reagents, which has great potential and promise for application. Current microfluidic cell spheroid formation chips can be divided into three main categories: (1) using emulsion technology, (2) utilizing U‐shaped microstructures and (3) utilizing microwells.^4^, ^5^ Among them, microwells‐based structures are more utilized due to their simplicity and ease of operation. However, the escape of spheroids from the microwells often influences the assay when high flow rates were used in the main channel.^6^

A large number of cell analysis studies rely on testing the response of cells to a range of reagent concentrations. Especially, concentration gradient tests are widely applied in drug screening. Microfluidic chips have the advantages of miniaturization, automation, high throughput, easy adjustment and precise control, and therefore play a unique advantage in concentration gradient studies.^7^

The most widely used microfluidic concentration gradient methods include tree networks and droplet microfluidics. Kim et al. coupled a tree‐like gradient generator design to an array of chambers that can be actively separated using valves.^8^ This structure has a relatively large device footprint and generates a large shear force. And the geometry of the tree structure determines the achievable gradient. Droplet microfluidics can generate large numbers of water‐in‐oil droplets at high rates. The contents of droplets can be adjusted by regulating the chemical composition of the aqueous solution at different inlets as well as the relative flow rate.^9^ However, the use of droplets brings complexity to droplet tracking and data collection. Therefore, various microfluidic techniques have been developed to divide samples into fixed positions.^10^, ^11^, ^12^, ^13^, ^14^ Based on the droplet generation chip, droplets containing samples of different concentrations can be obtained by gradually adjusting the composition of the formed droplets through dilution during the droplet formation process. Sun et al. formed fixed‐position droplet arrays with different sample concentrations by droplet dilution on a microfluidic device.^10^

Further, the construction of concentration gradients by liquid dilution methods appears to have more development as well as wider application because of simple structure, high throughput and low demand for peripherals. Watanabe et al. implemented a high‐throughput microreactor array with concentration gradients by simple structural design and liquid manipulation.^11^ Zhang et al. used 3D printing to design a simple microfluidic chip that enables the generation of concentration gradients for antibiotic susceptibility testing (AST) of *Escherichia coli*.^12^ Zeng et al. used syringes to manually create different levels of negative pressure environment inside the chip to control the generation of concentration gradients and to measure the gradient by the area of the droplet.^13^ Avesar et al. proposed a method for generating digital chemical gradients along 200 nL cell culture chambers and used computational and numerical models to predict the concentration in each chamber.^14^ They demonstrated that the concentration gradient can be controlled by flow parameters. However, it has not been successfully applied to the detection of cell spheroids with large incubation chamber.

In this study, we propose a concentration gradient generator for cell spheroid culture and drug screening. With the PDMS concave‐bottom microwell structure, cell spheroid formation is achieved inside the chip. Combined with microfluidic technology, we construct independent units with concentration gradients to achieve rapid response to dynamic gradients and long‐term stability of molecular gradients, and then use the chip for drug screening of cell spheroids.

## 2 RESULTS

### 2.1 Chip and experiment design

**FIGURE 1:**
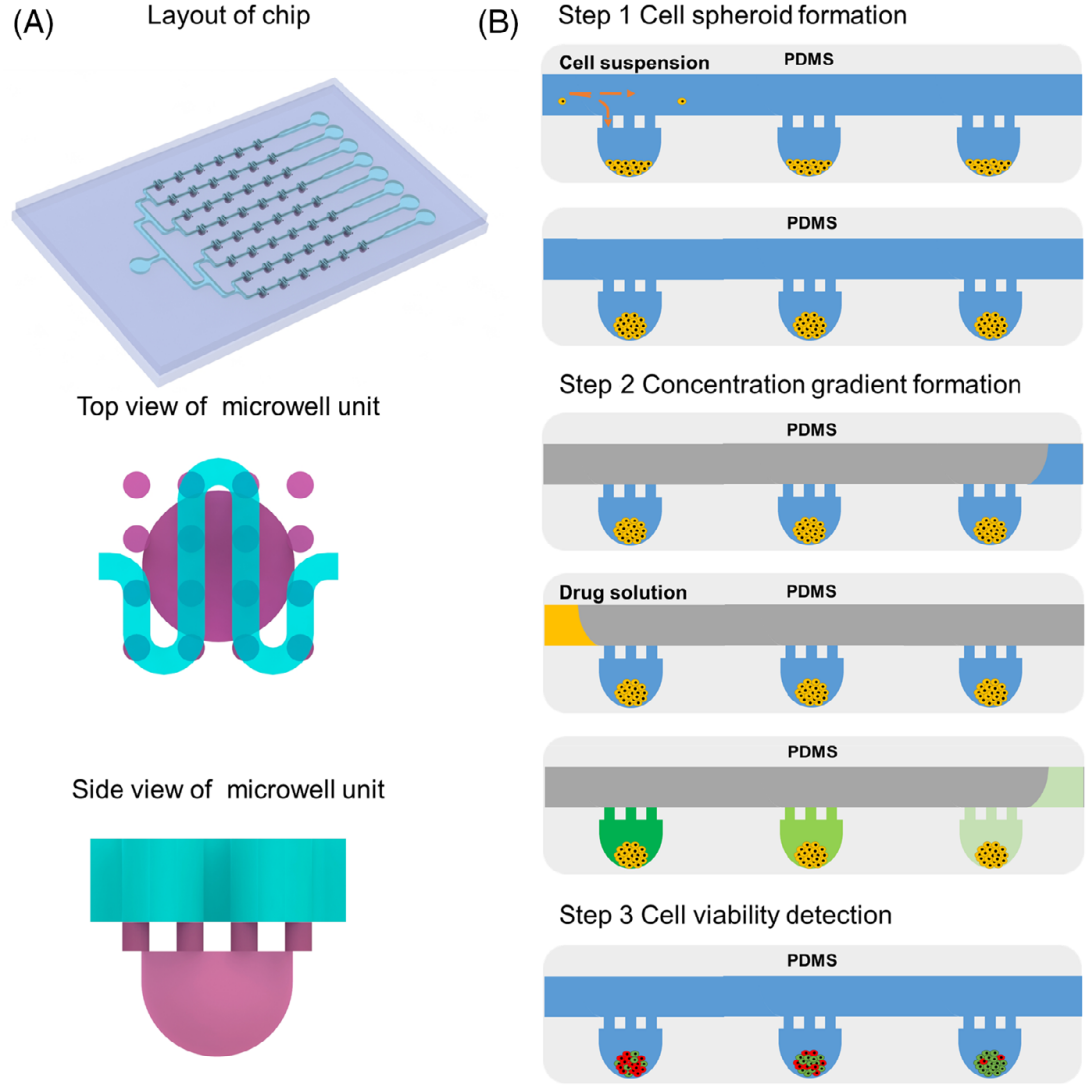
Schematic diagram of microfluidic concentration gradient chip and gradient formation process. (A) The layout of the microfluidic chip including the concave bottom microwell and S‐shaped channel connected by the through‐hole, insets show the top view and side views of the through‐hole together with microwell structure. (B) Schematic illustration of the procedure for the formation of a dye concentration gradient of cell load, spheroids formation and gradients implementation in the chip. Only three microwells are shown here for simplicity.

The 3D structure of the chip is shown in Figure 1A. We use PDMS material to build the overall structure of the chip. The chip consists of a three‐layer structure. There are six concave bottom microwells of 500 μm diameter in a row along each channel at the bottom layer. The microwells are connected to the upper layer channel by through‐holes of 100 μm diameter in the middle layer, distributed along the channel direction. The through‐holes have two functions including avoiding the escape of spheroids from microwells and cutting off the solution connection between the microchannel and the microwell when a section of solution flow over the microwells.

Figure 1B illustrates simplified process for performing the chip function. The process consists of the following steps. First, cells are loaded and partitioned in the microwells, followed by incubating to form cell spheroids. Then the culture medium in the microchannel is drained and a chemical solution at given volume followed by oil is infused into the microchannel. The solution flows successively over the microwells and is continually diluted by the medium in the microwells, resulting in the formation of concentration gradient in the microwells along the microchannel from inlet to outlet. The hydrophilic treatment of the PDMS surface allows the cells to preferentially aggregate in the microwells. Draining the medium from the channel before adding the chemical solution reduces the effect of channel liquid on the gradient efficacy. Meanwhile, the advance of the liquid can be clearly observed under the microscope as the channel has been previously emptied.

### 2.2 Simulation of through‐hole structure

To achieve microwell partitioning, we use through‐hole structures in the chip to maintain the stability of the medium in the microwell. Due to surface tension, when the medium is removed from the channel, the medium inside the microwell is lost along with it, and cell spheroid may escape. At this point, the presence of the through‐pore structure acts as a protection for the microwell contents, effectively stopping the loss of medium and attenuating the disturbance within the microwell. However, the structure may initially prevent liquids from entering the microwell.

We used a computational model to investigate the fluid entry and partitioning effect of the chip. The model included the complete geometry and the materials used were water and air. To study the effect of through‐hole shear liquid to achieve sufficient retention of liquid in the concave bottom microwells, the structure was set to be filled with liquid, and air entered through the inlet to carry away the liquid in the channel. For all simulations, the results had good convergence. In addition, there is no significant difference in the results after refining the mesh density.

**FIGURE 2:**
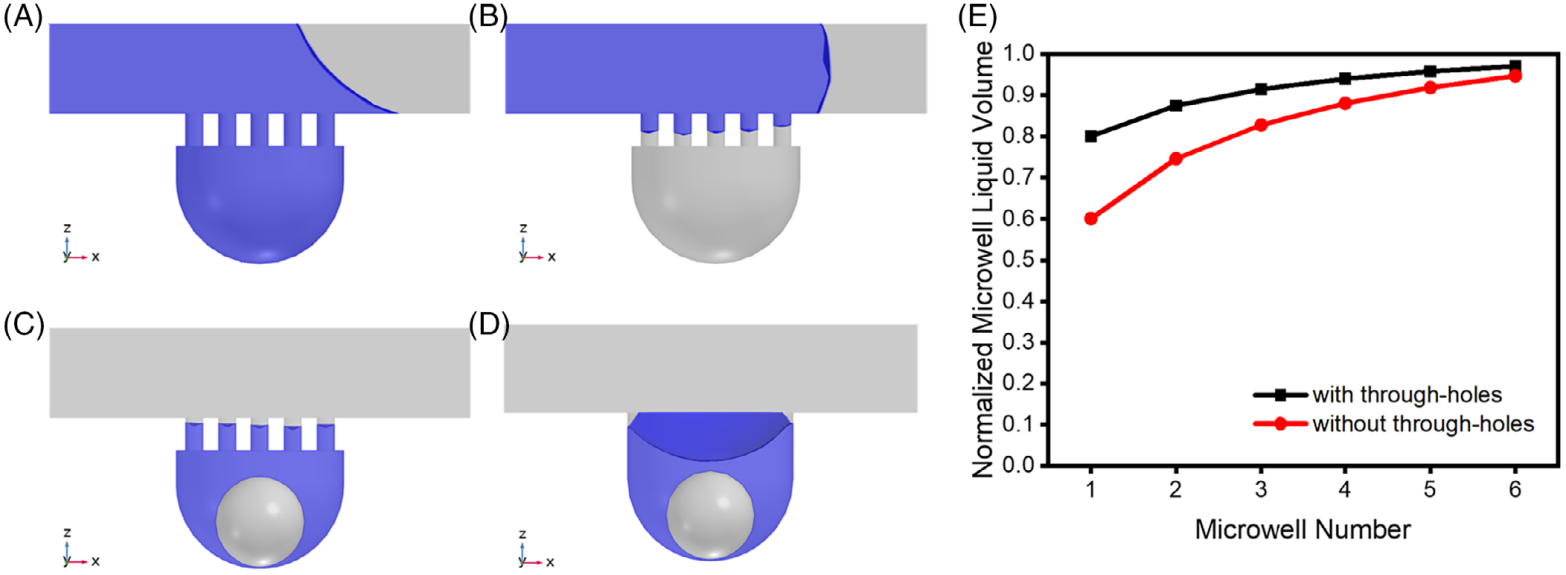
Simulation results of the effect of through‐hole structure. (A) A flow study when liquid enters through channel inlet. (B) A flow study while the chip does not have hydrophilic treatment or negative pressure environment. (C) A flow study when channel liquid exhausted from the chip. A solid ball is placed in the concave bottom microwell to represent the cell spheroid. (D) A flow study when channel liquid exhausted from the chip without through‐hole structure. (E) Comparison of liquid amount left in microwells with or without through‐hole structure.

Figure 2A–D shows the simulation results of the functional effects of fluid entry and partitioning effect of the chip, which was obtained through a two‐phase flow study. First, the liquid(blue) fills the concave bottom microwells and the flow channel (Figure 2A), which is achieved by the hydrophilic treatment of the PDMS surface as well as the negative pressure treatment. Without these treatments, liquid cannot enter the microwell (Figure 2B). Then air enters through the channel inlet to carry away the liquid in the channel, leaving as much liquid in the concave bottom microwell as possible due to the through‐hole structure (Figure 2C). The dark blue section in Figure 2D represents the loss of the solution. Figure 2E shows the amount of liquid left in the concave bottom microwell in the same situation without the through‐hole structure. We found that the amount of liquid left in the concave bottom microwell with the through‐hole structure was significantly more than that without the through‐hole structure, which helped to expand the gradient range and, at the same time, the through‐hole structure can avoid the loss of cell spheroids.

At the step of emptying the channel liquid, we also compared the functional effects of positive pressure operation and negative pressure operation. We found that negative pressure from the outlet provides a better liquid retention effect than pressure from the inlet. This is attributed to the fact that when positive pressure is applied from the inlet, the liquid in the microwell is simultaneously subject to compression forcing it out of the microwell while the channel liquid is under pressure.

In addition, the effects of different pore sizes of through‐hole membranes were compared. It was found that the 100 μm pore size membrane was able to achieve liquid retention. The 200 μm pore size membrane showed a loss of liquid in the microwell and was unable to achieve greater liquid retention (Figure S1). To maintain the function of the structure while enhancing the ease of cell inoculation, 100 μm pore size membranes were used.

### 2.3 Simulation and experimentation of gradient formation and modification

To study the concentration of the substance in the concave bottom microwells, mass transfer was studied by coupling the study to a dilute substance transfer physical interface using the convection–diffusion equation. No flux conditions were set on the wall. The inlet used water with a concentration of 1 mol/L as the drug delivery source, delivered at a velocity of 4 mm/s for 4 s. A physical field control grid was used. For all simulations, the results had good convergence. In addition, no significant differences in results were observed after refining the grid density.

**FIGURE 3:**
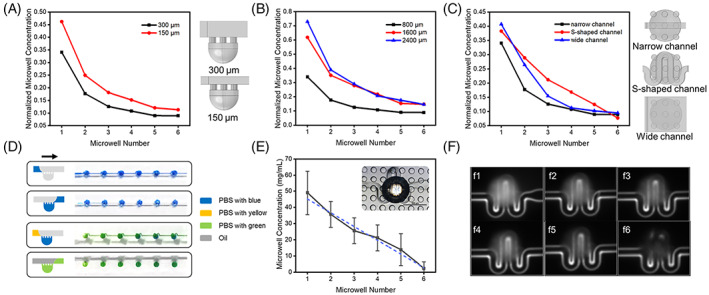
Simulation and experimental results of microfluidic concentration gradient chip. (A) Comparison of the concentration gradient of channel height, inset shows the side view of the structure. (B) Comparison of the concentration gradient of microwell spacing. (C) Comparison of the concentration gradient of different channel structures, inset shows the top view of the structure. (D) Schematic illustration of the procedure for the formation of a dye concentration gradient. (E) Quantification of microwell fluorescence intensity for S‐shaped channel. The experiment was repeated three times and six chambers were counted each time. (F) Fluorescence images of the results of microwell rhodamine B experiments.

Figure 3A–C shows the simulation results for mass transfer in the chip. The solute flows from the channel into the microwells, which persists while the liquid is connected to the microwells. To investigate the gradient effect of channel height, different flow channel heights were used, and the amount of substance concentration in each microwell is shown in Figure 3A. We found a similar trend in the concentration gradient of microwells whose channel heights were 150 and 300 μm, respectively. A greater range of concentration gradients was obtained for 150 μm height. This may be caused by the fact that the channel height close to the through‐hole diameter makes it easier for the solute to enter the microwells.

Also, varying distributions of microwells bring about differences in the gradient effect. To investigate the gradient effect of microwell distribution, different spacing was used to investigate the amount of substance concentration in each microwell. Results show that different microwell distributions lead to different concentration gradient outcomes (Figure 3B). The larger the microwell spacing, the larger the gradient range obtained. However, there is no great difference in the gradient range when the spacing is further increased (2400 μm). This suggests that the microwell distribution can be designed according to the gradient demand. But excessive spacing brings an increase in footprint and is not conducive to device miniaturization. Hence choosing the right spacing as needed is necessary.

Further, alterations in the channel shape also lead to modifications in the chip gradient effect. To investigate the gradient effect of channel shape, three different channel structures were investigated, S‐shaped, narrow channel, and wide channel, as shown in Figure 3C. We found that the gradients obtained with the S‐shaped flow channel structure were closer to linear, which may be related to the adequate mixing time. The multiple contacts of liquid between channel and microwell increases the mixing efficiency.

To visualize and quantify the gradients, we used dye and 62.5 mg/mL of rhodamine B mixed with PBS, respectively, to simulate the process of generating gradient concentrations on the S‐shaped channel chip (Figure 3D). The insert in Figure 3E is a physical view of the chip structure under the microscope. The microwell part appears to have a black outer ring and a bright centre due to the concave bottom structure. The fluorescent reagent rhodamine B diffuses into each chamber once it arrives at the inlet of the concave bottom microwells and contacts the liquid in the microwells through the through‐hole. The concentration decreases from front to back, resulting in a gradient of decreasing concentrations in the concave‐bottom microwells, ranging from 49.0 to 2.4 mg/mL (Figure 3E,F). We found that the obtained gradient trend is consistent with the simulation results (Figure 3C), indicating that the gradient effect can be predesigned by structure design and simulation. Similar experiment results were obtained by pigment experiments (Figure 3D).

### 2.4 Application of the chip in the cell spheroids drug assay

**FIGURE 4:**
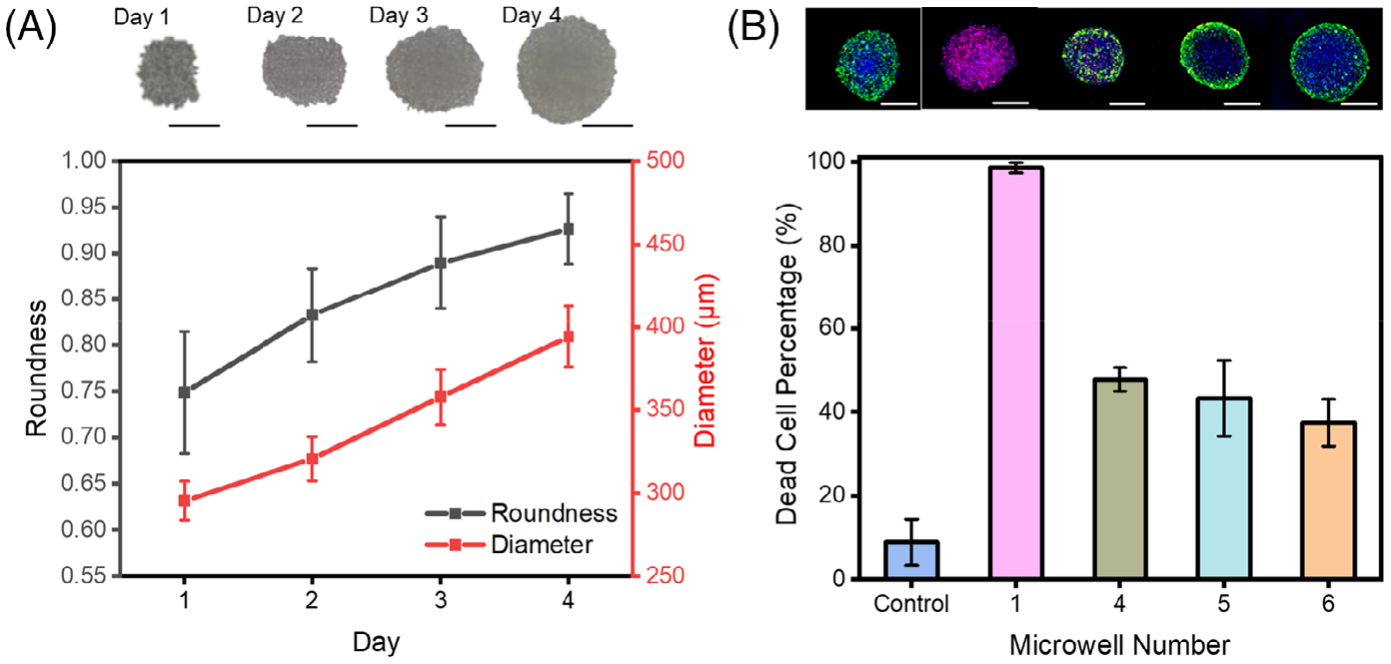
Photographs of (A) cell spheroid formation experiment and (B) drug concentration gradient experiment. The experiment was repeated three times. Scale bar: 200 μm.

We experimentally validated the formation of a gradient using improved parameters, where the entry channel is used for cell delivery as well as drug delivery. HepG2 cells entered the channel and fell into the concave bottom microwells through multiple 100 μm diameter through‐holes, where they clustered and grew into cell spheroids, as shown in Figure 4A. During 4 days' incubation, HepG2 spheroids formed and became bigger and rounder. Before the drug was added, the medium in the channel was discharged, and the cell spheroids and medium in the concave bottom microwell were retained due to the presence of the through‐holes.

In this experiment, we used the anticancer drug doxorubicin for the efficacy assessment of different concentrations in HepG2 cell spheroids. On day 5, the drug was added, and the solute entered the concave bottom microwell through the through‐hole and mixed with the medium left behind. After 2 days' drug treatment, differences in cell activity were observed (Figure 4B). Blank group had the least number of dead cells. High concentrations of microchambers exhibited very high cell mortality, while other concentrations entailed certain cellular damage. This illustrates the ability of the chip to be used to achieve drug gradients that can be utilized to estimate the appropriate range of drug action concentrations.

## 3 MATERIALS AND METHODS

### 3.1 Computational models

To understand the flow state of the liquid inside the chip and the effect of mixing of substances, a commercial finite element analysis (FEA) software COMSOL Multiphysics (version 5.6, COMSOL Inc.) was used for the analysis. In the simulation, a 3D model was constructed based on the design dimensions of the chip. The material used for the liquid is water and the material used for the gas is air. The two‐phase flow, level set transient study was performed with no‐slip boundary conditions, defined velocity at the inlet, and pressure boundary conditions at the outlet. To study the effect of the through‐hole on the retention of liquid in the concave bottom microwell, the structure was set to be filled with liquid, and air entered through the inlet of the channel taking away the liquid in the flow channel. To study the concentration of material in the concave bottom microwell, mass transfer was studied by coupling the study to a dilute material transfer physical interface using the convection–diffusion equation. No flux conditions were placed on the walls and the inlet used water at a concentration of 1 as the drug delivery source. A physical field control grid was used. For all simulations, the results had good convergence.

### 3.2 Design and fabrication of concentration gradient chip

The chip consists of three layers of PDMS structures bonded together, the upper PDMS (Dow Corning) channel structure, the middle through‐hole structure, and the lower concave bottom microwell structure, as shown in Figure 1A. PDMS structures were fabricated using a well‐established soft photolithography replica moulding process. Briefly, the master mould with positive relief features was fabricated by patterning a negative photoresist (SU‐83050, Kayaku) on a silicon wafer using a conventional photolithography technique. The mould was then silanized with Trichloro (1H,1H,2H, and 2H‐perfluorooctyl) silane (448931, Sigma‐Aldrich) in a desiccator at room temperature for 2 h to prevent undesired bonding between PDMS and the mould. the PDMS precursor was prepared by mixing base and curing agent in a 10:1 (w/w) ratio. The PDMS precursors were poured onto the fabricated moulds and then cured in an oven at 60°C for 4 h. A 1.4‐mm biopsy punch was used to punch holes at the inlet and outlet of the microchannel. The lower concave bottom microwell structure was obtained by scratch‐coating PDMS in a cylindrical PDMS structure made by a soft lithography process. The diameter and depth of the concave bottom microwell were 560 and 500 μm, respectively. Above the microwell are an array of through‐holes with aperture diameter, spacing, and height of 100, 200, and 110 μm, respectively. The three PDMS layers were bonded together by plasma treatment to obtain the overall structure, followed by hydrophilic treatment with F127 (P2443, Sigma‐Aldrich)..

### 3.3 Generation of concentration gradients

The three‐dimensional schematic is shown in Figure 1A. First, the medium was injected into the concave bottom microwell of the bottom layer through the channel, and HepG2 cells were also able to enter the concave bottom microwell through the channel and stayed inside the microwell, where cell aggregation occurred and grew into cell spheroids. Before adding drugs, the medium in the channel was emptied while retaining the medium in the concave bottom lumen as well as the cells. Afterward, a fixed volume of the drug was added and the drug moves to mix with the liquid in the concave bottom chamber and produce separate units.

To visualize the gradient profile, we used 62.5 mg/mL rhodamine B (71036314, Sinopharm Chemical Reagent Co., Ltd) and phosphate buffered saline (PBS pH 7.4 basic, Gibco) to model and visualize the process of generating gradient concentrations on the S‐shaped channel chip. The fluorescent reagent rhodamine B diffuses into each chamber once it arrives at the inlet of the concave bottom microwells and contacts the liquid in the microwells through the through‐hole. The concentration decreases from front to back, resulting in a gradient of decreasing concentrations in the concave‐bottom microwells. The process was performed using a pipette to avoid the use of complex instrumentation. The process can also be controlled automatically by a syringe pump. Fluorescence images of each concave well were taken using a fluorescence microscope and the concentration of fluorescein in each well was calculated from the standard curve.

### 3.4 Culture of cells

HepG2 cells were cultured in high glucose DMEM (Gibco) medium supplemented with 10% foetal bovine serum (FBS, Gibco). All cells were stored in T‐25 flasks according to standard culture procedures until collected for inoculation into microwells.

In drug experiment, we used 1.5 μL of 40 mg/mL doxorubicin (D1515, Sigma‐Aldrich) in each experiment.

In cell activity studies, live cells, as well as dead cells, were determined using the Dead Alive Dye LIVE/DEAD Cell Imaging Kit (R37601, Invitrogen) by incubation for 20 min. The cell nucleus dye Hoechst 33342 was used to help localize the cells. Fluorescent images were taken using a confocal microscope.

## 4 DISCUSSION AND CONCLUSION

The incidence of early stage cancer has increased dramatically worldwide since 1990. Individual differences require individualized cancer treatment, and appropriate in vitro models are important for early cancer treatment. The cell spheroid model exhibits characteristics similar to those of in vivo tumour tissue. These characteristics make cell spheroids a more suitable tool for in vitro drug screening analysis than 2D monolayer cell cultures.

The chip we present here is useful for the in situ analysis of cell spheroid models. The chip avoids the loss of cell spheroids and provides a concentration gradients. Using numerical simulations, we showed that the value of shear stress in the wells of the chip is significantly low and will not affect the function and viability of cell spheroids. Using three independent gradient formation experiments, we find that the gradient formation matches the analytical model with high accuracy, demonstrating that the gradients can be reproduced experimentally. The gradient profile can be modified by adjusting the structure parameters including microchannel height, the space between wells and microchannel shape. Finally, we demonstrate the ability of the chip to study the effects of drug on the cell spheroids. Compared with microtitre plates, this chip can reduce the culture microwell volume by multiple orders of magnitude, thus greatly improving the sensitivity for cell analysis applications. In addition, microwell volumes can be easily customized by changing the concave bottom microwell size, resulting in different‐sized cell spheroids.

The low‐cost and simple approach is promising for high‐throughput and standardized anti‐cancer drug screening.

## AUTHOR CONTRIBUTIONS

**Panhui Yang:** Methodology, validation, investigation, data curation, writing—original draft preparation. **Lei Wu:** Conceptualization, methodology, writing—review and editing. **Guoyuan Zhang:** Formal analysis, investigation. **Yuqing Ge:** Validation. **Ting Liu:** Visualization. **Hongju Mao:** Resources, supervision. **Jianlong Zhao:** Project administration, funding acquisition.

## Supporting information


**Figure S1.** Comparison of the liquid retention effects of through‐hole membranes with different pore sizes. (a) Photographs of chip with 100 μm pore size through‐hole membranes. (b) Photographs of chip with 200 μm pore size through‐hole membranes. The blue dye illustrates the advance of the liquid. Scale bar: 250 μm.

## Data Availability

The data that support the findings of this study are available from the corresponding author upon reasonable request.
