# Supplementary material for: Cell spheroids culture array with modifiable chemical gradients
Source: Cell Prolif. 2023 May 17;56(5):e13473. doi: 10.1111/cpr.13473 (PMC10212701; doi:10.1111/cpr.13473)
Supplement: Supplementary file 1 — Figure S1. Comparison of the liquid retention effects of through‐hole membranes with different pore sizes. (a) Photographs of chip with 100 μm pore size through‐hole membranes. (b) Photographs of chip with 200 μm pore size through‐hole membranes. The blue dye illustrates the advance of the liquid. Scale bar: 250 μm. [file CPR-56-e13473-s001.docx]

Supplementary information

**Cell Spheroids Culture Array with Modifiable Chemical Gradients**

*Panhui Yang, Lei Wu*, Guoyuan Zhang, Yuqing Ge, Ting Liu, Xiyao Peng, Hongju Mao*, Jianlong Zhao**

Supplementary Figures


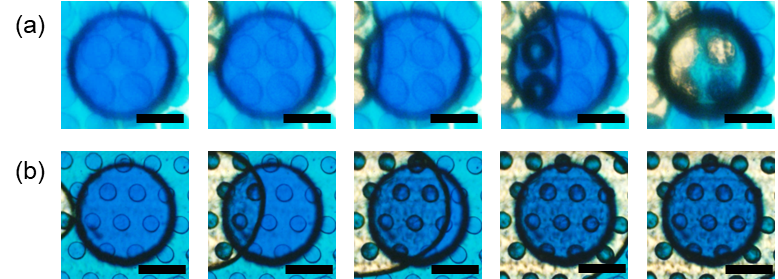


**Fig. S1 Comparison of the liquid retention effects of through-hole membranes with different pore sizes. (a) Photographs of chip with 100um pore size through-hole membranes. (b) Photographs of chip with 200um pore size through-hole membranes. The blue dye illustrates the advance of the liquid. Scale bar: 250um.**
